# Infectious Diseases and Sexual Coercion^1^

**DOI:** 10.3201/eid1011.040623_05

**Published:** 2004-11

**Authors:** Consuelo M. Beck-Sague, Adrienne Wulfsohn, Chris Beyrer, Elizabeth Unger, Carolyn Black

**Affiliations:** * Centers for Disease Control and Prevention, Atlanta, Georgia, USA;; † PROAct, Johannesburg, South Africa;; ‡ Johns Hopkins University Bloomberg School of Public Health, Baltimore, Maryland, USA

**Keywords:** sexual assault, rape, sexual abuse, trafficking, HIV, sexually transmitted diseases

## South Africa: Sexual Assault and STIs

South Africa's HIV prevalence (20.1%), particularly in Gauteng Province (29.8%), is among the highest in the world. Incidence of rape, defined as genital, anal, or oral penetration by a part of the assailant's body or with an object, is high; most South African women have survived more than one rape. From 1998 to 2002, a facility in Johannesburg offered antiretroviral postexposure prophylaxis to sexual assault survivors seen within 72 hours of assault. Of 858 examined, 65% were assaulted by multiple assailants; 14% and 22% were HIV-positive in the first and final years of the study (1998 and 2002), respectively. Of 435 who received antiretroviral postexposure prophylaxis, 173 returned for repeat HIV testing at 6 weeks; HIV infection was diagnosed in 1 (0.2%) patient 8 months after the rape (1 month after symptoms consistent with acute retroviral infection). This patient was a mentally challenged 14-year-old who did not complete postexposure prophylaxis, was HIV-negative at 6 weeks, and may have been subsequently exposed. Of 25 seronegative patients who sought treatment >72 hours after assault, 9 returned for follow-up examination; 1 (4%) seroconverted at 6 weeks. This patient was a 16-year-old without other risk factors. The South African government agreed to make HIV postexposure prophylaxis broadly available in 2002 but has not yet done so.

## Human Trafficking

Defined as transport of a person for work or services within or across national borders through force, deception, or abuse of authority, trafficking annually victimizes millions, trapping them in virtual slavery as commercial sex workers or domestic or sweatshop workers. This traffic disproportionately affects women. Source countries of trafficked women include Burma, Thailand, Russia, Laos, China, and the Philippines; destination countries include Thailand, China, Cambodia, India, Russia, Sweden, the United States, and the European Union. Countries with domestic sex worker trafficking include China, India, Thailand, Cambodia, and Burma. Health threats to trafficked women include HIV, other STIs, severe mental health problems, and low access to preventive healthcare. The HIV rate of trafficked Burmese commercial sex workers is more than twice that of Thai commercial sex workers. Burma's trafficking disproportionately affects persons of Shan ethnicity, who have been made vulnerable by civil war, discrimination, and poverty. Effective interventions include recognizing that trafficked women are crime victims who deserve special consideration; avoiding trafficked women's repatriation; trafficker prosecution; and partnerships between health, civil, and security officials.

## Child Sexual Abuse

Child sexual abuse is common; 42% of U.S. adult women report sexual victimization by an adult or older minor during childhood. STIs diagnostic for child sexual abuse (e.g., *Neisseria gonorrhoeae* or *Chlamydia trachomatis*, diagnosed by culture, which is less sensitive than nucleic acid amplification testing) are uncommon. The relationship of genital human papillomavirus infection, the most common STI, to child sexual abuse, remains unclear. Genital swab and urine samples from minors evaluated for sexual abuse were tested by using nucleic acid amplification for *N. gonorrhoeae*, *C. trachomatis*, and human papillomavirus. Of 439 evaluated, 5.2% had positive nucleic acid amplification test results (vs. 3.5% with positive cultures) for *N. gonorrhoeae* or *C. trachomatis*; 13% (17% of abused minors, but none of 16 children unlikely to have been abused) had human papillomavirus detected by nucleic acid amplification tests. Nucleic acid amplification tests increased the proportion of child sexual abuse survivors with a diagnosis of *N. gonorrhoeae* or *C. trachomatis* infection. Positive nucleic acid amplification tests for human papillomavirus were strongly associated with child sexual abuse.

